# 3,4-Dimethyl­pyrano[2,3-*c*]pyrazol-6(2*H*)-one

**DOI:** 10.1107/S1600536812012779

**Published:** 2012-03-28

**Authors:** Bilal Shahid, Muhammad Zia-ur-Rehman, Muhammad Nadeem Arshad, Rabia Nazir, Ertan Şahin

**Affiliations:** a Department of Chemistry, GC University, Lahore 54000, Pakistan; b Applied Chemistry Research Centre, Pakistan Council of Scientific & Industrial Research Laboratories Complex, Lahore 54600, Pakistan; c Department of Chemistry, University of Gujrat (H. H. Campus), Gujrat 57000, Pakistan; d Department of Chemistry, Faculty of Science, Atatürk University, 25240 Erzurum, Turkey

## Abstract

The asymmetric unit of the title compound, C_8_H_8_N_2_O_2_, comprises two independent mol­ecules in both of which, all non-H atoms lie in a common plane (r.m.s. deviation = 0.014 and 0.017 Å). In the crystal, N—H⋯O hydrogen bonds connect the mol­ecules into zigzag chains running along [10-1]. Weak C—H⋯O inter­actions connect the chains into an infinite network.

## Related literature

For related structures, see: Ahmad *et al.* (2011 ▶); Ramsay & Steel (1985 ▶).
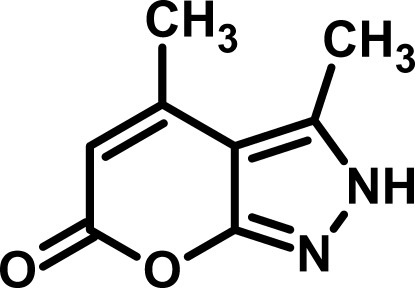


## Experimental

### 

#### Crystal data

C_8_H_8_N_2_O_2_
*M* *_r_* = 164.16
Monoclinic, 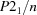

*a* = 13.6219 (3) Å
*b* = 6.8766 (2) Å
*c* = 16.2369 (4) Å
β = 96.091 (2)°
*V* = 1512.36 (7) Å^3^
*Z* = 8
Mo *K*α radiation
μ = 0.11 mm^−1^
*T* = 296 K
0.30 × 0.18 × 0.11 mm

#### Data collection

Bruker Kappa APEXII CCD diffractometer
Absorption correction: multi-scan (*SADABS*; Bruker, 2007 ▶) *T*_min_ = 0.970, *T*_max_ = 0.989
16831 measured reflections
3770 independent reflections
2654 reflections with *I* > 2σ(*I*)
*R*_int_ = 0.031

#### Refinement

*R*[*F* ^2^ > 2σ(*F* ^2^)] = 0.041
*wR*(*F* ^2^) = 0.117
*S* = 1.02
3770 reflections
228 parameters
H atoms treated by a mixture of independent and constrained refinement
Δρ_max_ = 0.23 e Å^−3^
Δρ_min_ = −0.16 e Å^−3^

### 

Data collection: *APEX2* (Bruker, 2007 ▶); cell refinement: *SAINT* (Bruker, 2007 ▶); data reduction: *SAINT*; program(s) used to solve structure: *SHELXS97* (Sheldrick, 2008 ▶); program(s) used to refine structure: *SHELXL97* (Sheldrick, 2008 ▶); molecular graphics: *ORTEP-3 for Windows* (Farrugia, 1997 ▶) and *PLATON* (Spek, 2009 ▶); software used to prepare material for publication: *WinGX* (Farrugia, 1999 ▶) and *PLATON*.

## Supplementary Material

Crystal structure: contains datablock(s) I, global. DOI: 10.1107/S1600536812012779/bt5857sup1.cif

Structure factors: contains datablock(s) I. DOI: 10.1107/S1600536812012779/bt5857Isup2.hkl

Supplementary material file. DOI: 10.1107/S1600536812012779/bt5857Isup3.cml

Additional supplementary materials:  crystallographic information; 3D view; checkCIF report

## Figures and Tables

**Table 1:** Hydrogen-bond geometry (Å, °)

*D*—H⋯*A*	*D*—H	H⋯*A*	*D*⋯*A*	*D*—H⋯*A*
N4—H4*N*⋯O2^i^	0.91 (2)	1.91 (2)	2.7860 (17)	160.9 (17)
N2—H2*N*⋯O4^ii^	0.912 (19)	1.984 (19)	2.8872 (18)	170.4 (17)
C2—H2⋯O2^iii^	0.93	2.49	3.4082 (19)	171
C7—H7*A*⋯O3^ii^	0.96	2.54	3.458 (2)	159
C10—H10⋯O4^iv^	0.93	2.45	3.3563 (18)	164

Symmetry codes: (i) 
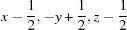
; (ii) 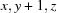; (iii) 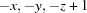; (iv) 
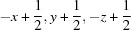
.

**Fig. 1.**
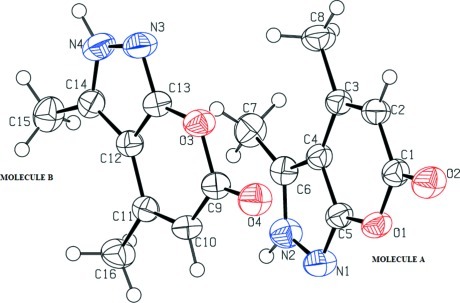
The labelled molecular structure of (I) showing molecules A and B with 50% displacement ellipsoids.

**Fig. 2.**
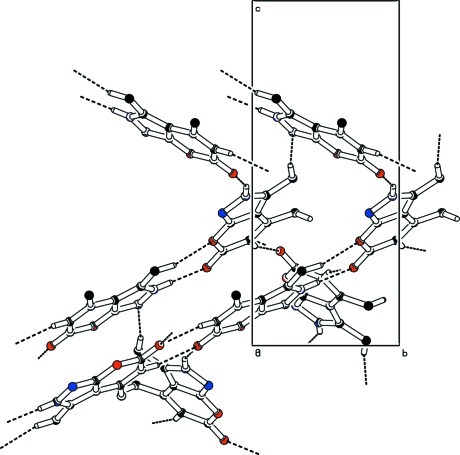
A perspective view showing hydrogen bond interactions drawn as dashed lines.

## supplementary crystallographic information

### Comment

In continuation of our work on the synthesis and biological evaluation of various heterocyclic compounds, we, herein report the crystal structure of the title compound (I) Fig. 1.

Structure of the title compound is closely related to already published structures of 3,4-dimethyl-1-(2-pyridyl)pyrano(2,3-*c*)pyrazol-6 (1*H*)-one (Ramsay & Steel, 1985) and 3,4-dimethyl-1-phenylpyrano [2,3-*c*]pyrazol-6(1*H*)-one (Ahmad *et al.*, 2011). There are two asymmetric molecules per unit cell and each molecule comprises of one pyranone ring fused with pyrazole ring. N-H···O hydrogen bonding interactions connect the molecules to zig-zag chains running along [1 0 1]. In addition, weak intermolecular C—H···O interactions connect these infinite chains to a three dimesional network (Table 1, Fig. 2).

### Experimental

A mixture of hydrazine hydrate (1.0 mmole), ethyl acetoacetate (2.0 mmoles) was heated for one hour at 120°C followed by cooling to room temperature. The contents were triturated with diethyl ether, filtered and the residue obtained was crystallized from acetic acid. M.p. 246°C; Yield (87%).

### Refinement

All H-atoms bonded to C were positioned with idealized geometry with C—H = 0.93 Å for aromatic and C—H = 0.96 Å for methyl groups and were refined using a riding model with *U*_iso_(H) = 1.2 *U*_eq_(C) for aromatic and *U*_iso_(H) = 1.5 *U*_eq_(C) for methyl carbon atoms. The coordinates of the H atoms bonded to N were refined with *U*_iso_(H) = 1.2 *U*_eq_(N).

### Crystal data

**Table:**

C_8_H_8_N_2_O_2_	*F*(000) = 688
*M**_r_* = 164.16	*D*_x_ = 1.443 Mg m^−^^3^
Monoclinic, *P*2_1_/*n*	Melting point: 518 K
Hall symbol: -P 2yn	Mo *K*α radiation, λ = 0.71073 Å
*a* = 13.6219 (3) Å	Cell parameters from 5051 reflections
*b* = 6.8766 (2) Å	θ = 2.5–27.8°
*c* = 16.2369 (4) Å	µ = 0.11 mm^−^^1^
β = 96.091 (2)°	*T* = 296 K
*V* = 1512.36 (7) Å^3^	Needle, yellow
*Z* = 8	0.30 × 0.18 × 0.11 mm

### Data collection

**Table:**

Bruker Kappa APEXII CCD diffractometer	3770 independent reflections
Radiation source: fine-focus sealed tube	2654 reflections with *I* > 2σ(*I*)
Graphite monochromator	*R*_int_ = 0.031
φ and ω scans	θ_max_ = 28.3°, θ_min_ = 1.9°
Absorption correction: multi-scan (*SADABS*; Bruker, 2007)	*h* = −18→18
*T*_min_ = 0.970, *T*_max_ = 0.989	*k* = −8→9
16831 measured reflections	*l* = −21→18

### Refinement

**Table:**

Refinement on *F*^2^	Secondary atom site location: difference Fourier map
Least-squares matrix: full	Hydrogen site location: inferred from neighbouring sites
*R*[*F*^2^ > 2σ(*F*^2^)] = 0.041	H atoms treated by a mixture of independent and constrained refinement
*wR*(*F*^2^) = 0.117	*w* = 1/[σ^2^(*F*_o_^2^) + (0.0553*P*)^2^ + 0.3127*P*] where *P* = (*F*_o_^2^ + 2*F*_c_^2^)/3
*S* = 1.02	(Δ/σ)_max_ < 0.001
3770 reflections	Δρ_max_ = 0.23 e Å^−^^3^
228 parameters	Δρ_min_ = −0.16 e Å^−^^3^
0 restraints	Extinction correction: *SHELXL97* (Sheldrick, 2008), Fc^*^=kFc[1+0.001xFc^2^λ^3^/sin(2θ)]^-1/4^
Primary atom site location: structure-invariant direct methods	Extinction coefficient: 0.0060 (10)

### Special details

**Table:**

Geometry. All s.u.'s (except the s.u. in the dihedral angle between two l.s. planes) are estimated using the full covariance matrix. The cell s.u.'s are taken into account individually in the estimation of s.u.'s in distances, angles and torsion angles; correlations between s.u.'s in cell parameters are only used when they are defined by crystal symmetry. An approximate (isotropic) treatment of cell s.u.'s is used for estimating s.u.'s involving l.s. planes.
Refinement. Refinement of F^2^ against ALL reflections. The weighted R-factor wR and goodness of fit S are based on F^2^, conventional R-factors R are based on F, with F set to zero for negative F^2^. The threshold expression of F^2^ > 2σ(F^2^) is used only for calculating R-factors(gt) etc. and is not relevant to the choice of reflections for refinement. R-factors based on F^2^ are statistically about twice as large as those based on F, and R- factors based on ALL data will be even larger.

### Fractional atomic coordinates and isotropic or equivalent isotropic displacement parameters (Å^2^)

**Table:**

	*x*	*y*	*z*	*U*_iso_*/*U*_eq_	
O1	0.14791 (7)	0.41930 (15)	0.44365 (6)	0.0399 (3)	
O2	0.13549 (8)	0.13137 (18)	0.50096 (7)	0.0512 (3)	
O3	0.00479 (8)	0.23381 (15)	0.19680 (7)	0.0417 (3)	
O4	0.14510 (8)	0.19091 (16)	0.27386 (7)	0.0493 (3)	
N1	0.14966 (10)	0.7225 (2)	0.38066 (8)	0.0445 (3)	
N2	0.07651 (10)	0.8288 (2)	0.33725 (9)	0.0446 (3)	
N3	−0.14217 (10)	0.2981 (2)	0.11578 (9)	0.0480 (4)	
N4	−0.17201 (10)	0.4537 (2)	0.06663 (9)	0.0475 (4)	
C1	0.09219 (11)	0.2599 (2)	0.46042 (9)	0.0372 (3)	
C2	−0.01109 (11)	0.2563 (2)	0.43051 (9)	0.0375 (3)	
H2	−0.0480	0.1485	0.4431	0.045*	
C3	−0.05750 (10)	0.4004 (2)	0.38520 (9)	0.0341 (3)	
C4	0.00154 (10)	0.5642 (2)	0.36819 (8)	0.0333 (3)	
C5	0.10172 (10)	0.5654 (2)	0.39826 (9)	0.0351 (3)	
C6	−0.01200 (11)	0.7428 (2)	0.32825 (9)	0.0375 (3)	
C7	−0.10067 (12)	0.8367 (3)	0.28407 (10)	0.0487 (4)	
H7A	−0.0840	0.9652	0.2671	0.073*	
H7B	−0.1518	0.8446	0.3204	0.073*	
H7C	−0.1236	0.7610	0.2362	0.073*	
C8	−0.16479 (11)	0.3900 (3)	0.35436 (11)	0.0473 (4)	
H8A	−0.1921	0.2711	0.3729	0.071*	
H8B	−0.1724	0.3937	0.2949	0.071*	
H8C	−0.1987	0.4986	0.3755	0.071*	
C9	0.09864 (11)	0.2979 (2)	0.22481 (9)	0.0364 (3)	
C10	0.13176 (10)	0.4804 (2)	0.19482 (9)	0.0368 (3)	
H10	0.1960	0.5196	0.2121	0.044*	
C11	0.07528 (10)	0.5986 (2)	0.14298 (9)	0.0332 (3)	
C12	−0.02244 (10)	0.5304 (2)	0.11579 (8)	0.0333 (3)	
C13	−0.05254 (11)	0.3506 (2)	0.14382 (9)	0.0363 (3)	
C14	−0.10441 (11)	0.5942 (2)	0.06431 (9)	0.0380 (3)	
C15	−0.12391 (13)	0.7747 (3)	0.01454 (11)	0.0509 (4)	
H15A	−0.1839	0.7596	−0.0217	0.076*	
H15B	−0.0700	0.7980	−0.0178	0.076*	
H15C	−0.1304	0.8828	0.0510	0.076*	
C16	0.11158 (13)	0.7903 (2)	0.11518 (11)	0.0481 (4)	
H16A	0.1782	0.8107	0.1394	0.072*	
H16B	0.0702	0.8923	0.1324	0.072*	
H16C	0.1097	0.7911	0.0559	0.072*	
H2N	0.0908 (14)	0.948 (3)	0.3166 (11)	0.058*	
H4N	−0.2329 (15)	0.450 (3)	0.0380 (11)	0.058*	

### Atomic displacement parameters (Å^2^)

**Table:**

	*U*^11^	*U*^22^	*U*^33^	*U*^12^	*U*^13^	*U*^23^
O1	0.0295 (5)	0.0439 (6)	0.0448 (6)	0.0013 (4)	−0.0031 (4)	0.0031 (5)
O2	0.0412 (6)	0.0510 (7)	0.0590 (7)	0.0083 (5)	−0.0059 (5)	0.0141 (6)
O3	0.0400 (6)	0.0337 (5)	0.0503 (6)	−0.0062 (4)	−0.0005 (5)	0.0064 (5)
O4	0.0461 (7)	0.0435 (6)	0.0568 (7)	0.0067 (5)	−0.0016 (5)	0.0123 (5)
N1	0.0365 (7)	0.0427 (7)	0.0533 (8)	−0.0043 (6)	0.0002 (6)	0.0018 (6)
N2	0.0455 (8)	0.0362 (7)	0.0518 (8)	−0.0023 (6)	0.0037 (6)	0.0039 (6)
N3	0.0368 (7)	0.0496 (8)	0.0561 (9)	−0.0102 (6)	−0.0017 (6)	0.0001 (7)
N4	0.0332 (7)	0.0567 (9)	0.0504 (8)	−0.0009 (6)	−0.0051 (6)	−0.0039 (7)
C1	0.0344 (8)	0.0406 (8)	0.0361 (8)	0.0032 (6)	0.0014 (6)	0.0002 (6)
C2	0.0318 (7)	0.0398 (8)	0.0409 (8)	−0.0023 (6)	0.0028 (6)	0.0035 (6)
C3	0.0289 (7)	0.0405 (8)	0.0330 (7)	0.0008 (6)	0.0037 (6)	−0.0025 (6)
C4	0.0305 (7)	0.0368 (7)	0.0323 (7)	0.0015 (6)	0.0022 (6)	−0.0013 (6)
C5	0.0307 (7)	0.0387 (8)	0.0352 (8)	0.0001 (6)	0.0009 (6)	−0.0020 (6)
C6	0.0387 (8)	0.0369 (8)	0.0371 (8)	0.0023 (6)	0.0044 (6)	−0.0008 (6)
C7	0.0493 (10)	0.0450 (9)	0.0510 (10)	0.0092 (7)	0.0017 (8)	0.0084 (7)
C8	0.0304 (8)	0.0539 (10)	0.0564 (10)	−0.0017 (7)	−0.0001 (7)	0.0063 (8)
C9	0.0339 (8)	0.0348 (8)	0.0404 (8)	0.0027 (6)	0.0035 (6)	−0.0002 (6)
C10	0.0278 (7)	0.0380 (8)	0.0443 (8)	−0.0033 (6)	0.0021 (6)	−0.0009 (6)
C11	0.0333 (7)	0.0318 (7)	0.0353 (7)	−0.0013 (6)	0.0067 (6)	−0.0027 (6)
C12	0.0325 (7)	0.0327 (7)	0.0351 (8)	0.0012 (6)	0.0051 (6)	−0.0025 (6)
C13	0.0333 (8)	0.0367 (8)	0.0386 (8)	−0.0028 (6)	0.0023 (6)	−0.0015 (6)
C14	0.0351 (8)	0.0434 (8)	0.0352 (8)	0.0053 (7)	0.0022 (6)	−0.0046 (6)
C15	0.0498 (10)	0.0539 (10)	0.0477 (10)	0.0141 (8)	−0.0013 (8)	0.0043 (8)
C16	0.0486 (10)	0.0381 (9)	0.0577 (10)	−0.0075 (7)	0.0059 (8)	0.0065 (7)

### Geometric parameters (Å, º)

**Table:**

O1—C5	1.3603 (17)	C6—C7	1.485 (2)
O1—C1	1.3768 (18)	C7—H7A	0.9600
O2—C1	1.2165 (18)	C7—H7B	0.9600
O3—C13	1.3609 (18)	C7—H7C	0.9600
O3—C9	1.3828 (18)	C8—H8A	0.9600
O4—C9	1.2122 (18)	C8—H8B	0.9600
N1—C5	1.3094 (19)	C8—H8C	0.9600
N1—N2	1.3695 (19)	C9—C10	1.436 (2)
N2—C6	1.337 (2)	C10—C11	1.350 (2)
N2—H2N	0.912 (19)	C10—H10	0.9300
N3—C13	1.3076 (19)	C11—C12	1.4361 (19)
N3—N4	1.370 (2)	C11—C16	1.495 (2)
N4—C14	1.338 (2)	C12—C14	1.393 (2)
N4—H4N	0.91 (2)	C12—C13	1.394 (2)
C1—C2	1.439 (2)	C14—C15	1.489 (2)
C2—C3	1.351 (2)	C15—H15A	0.9600
C2—H2	0.9300	C15—H15B	0.9600
C3—C4	1.428 (2)	C15—H15C	0.9600
C3—C8	1.495 (2)	C16—H16A	0.9600
C4—C6	1.392 (2)	C16—H16B	0.9600
C4—C5	1.3998 (19)	C16—H16C	0.9600
			
C5—O1—C1	117.54 (11)	C3—C8—H8B	109.5
C13—O3—C9	117.92 (11)	H8A—C8—H8B	109.5
C5—N1—N2	101.55 (12)	C3—C8—H8C	109.5
C6—N2—N1	114.76 (13)	H8A—C8—H8C	109.5
C6—N2—H2N	125.6 (12)	H8B—C8—H8C	109.5
N1—N2—H2N	119.7 (12)	O4—C9—O3	114.99 (13)
C13—N3—N4	101.29 (13)	O4—C9—C10	126.33 (14)
C14—N4—N3	114.69 (13)	O3—C9—C10	118.68 (13)
C14—N4—H4N	126.9 (12)	C11—C10—C9	123.95 (13)
N3—N4—H4N	118.4 (12)	C11—C10—H10	118.0
O2—C1—O1	116.08 (13)	C9—C10—H10	118.0
O2—C1—C2	124.78 (15)	C10—C11—C12	116.31 (13)
O1—C1—C2	119.13 (13)	C10—C11—C16	122.29 (14)
C3—C2—C1	123.74 (14)	C12—C11—C16	121.39 (13)
C3—C2—H2	118.1	C14—C12—C13	103.33 (13)
C1—C2—H2	118.1	C14—C12—C11	137.69 (14)
C2—C3—C4	116.33 (13)	C13—C12—C11	118.98 (13)
C2—C3—C8	122.12 (14)	N3—C13—O3	120.62 (13)
C4—C3—C8	121.55 (13)	N3—C13—C12	115.28 (14)
C6—C4—C5	103.48 (13)	O3—C13—C12	124.10 (13)
C6—C4—C3	137.45 (14)	N4—C14—C12	105.41 (13)
C5—C4—C3	119.05 (13)	N4—C14—C15	122.24 (14)
N1—C5—O1	120.99 (13)	C12—C14—C15	132.35 (15)
N1—C5—C4	114.79 (13)	C14—C15—H15A	109.5
O1—C5—C4	124.21 (13)	C14—C15—H15B	109.5
N2—C6—C4	105.43 (13)	H15A—C15—H15B	109.5
N2—C6—C7	122.57 (14)	C14—C15—H15C	109.5
C4—C6—C7	132.00 (14)	H15A—C15—H15C	109.5
C6—C7—H7A	109.5	H15B—C15—H15C	109.5
C6—C7—H7B	109.5	C11—C16—H16A	109.5
H7A—C7—H7B	109.5	C11—C16—H16B	109.5
C6—C7—H7C	109.5	H16A—C16—H16B	109.5
H7A—C7—H7C	109.5	C11—C16—H16C	109.5
H7B—C7—H7C	109.5	H16A—C16—H16C	109.5
C3—C8—H8A	109.5	H16B—C16—H16C	109.5
			
C5—N1—N2—C6	−0.04 (18)	C3—C4—C6—C7	−0.6 (3)
C13—N3—N4—C14	0.15 (18)	C13—O3—C9—O4	177.73 (13)
C5—O1—C1—O2	179.84 (13)	C13—O3—C9—C10	−1.88 (19)
C5—O1—C1—C2	−0.76 (19)	O4—C9—C10—C11	−176.82 (15)
O2—C1—C2—C3	−179.65 (15)	O3—C9—C10—C11	2.8 (2)
O1—C1—C2—C3	1.0 (2)	C9—C10—C11—C12	−1.7 (2)
C1—C2—C3—C4	−0.8 (2)	C9—C10—C11—C16	178.13 (14)
C1—C2—C3—C8	179.23 (14)	C10—C11—C12—C14	−179.24 (16)
C2—C3—C4—C6	−177.72 (16)	C16—C11—C12—C14	0.9 (3)
C8—C3—C4—C6	2.3 (3)	C10—C11—C12—C13	0.0 (2)
C2—C3—C4—C5	0.4 (2)	C16—C11—C12—C13	−179.86 (14)
C8—C3—C4—C5	−179.65 (14)	N4—N3—C13—O3	179.70 (13)
N2—N1—C5—O1	−178.64 (13)	N4—N3—C13—C12	−0.08 (17)
N2—N1—C5—C4	0.29 (17)	C9—O3—C13—N3	−179.50 (13)
C1—O1—C5—N1	179.23 (13)	C9—O3—C13—C12	0.3 (2)
C1—O1—C5—C4	0.4 (2)	C14—C12—C13—N3	−0.02 (17)
C6—C4—C5—N1	−0.42 (17)	C11—C12—C13—N3	−179.50 (13)
C3—C4—C5—N1	−179.09 (13)	C14—C12—C13—O3	−179.79 (13)
C6—C4—C5—O1	178.48 (13)	C11—C12—C13—O3	0.7 (2)
C3—C4—C5—O1	−0.2 (2)	N3—N4—C14—C12	−0.16 (18)
N1—N2—C6—C4	−0.21 (18)	N3—N4—C14—C15	−179.68 (14)
N1—N2—C6—C7	179.12 (14)	C13—C12—C14—N4	0.10 (15)
C5—C4—C6—N2	0.35 (15)	C11—C12—C14—N4	179.43 (16)
C3—C4—C6—N2	178.62 (16)	C13—C12—C14—C15	179.54 (16)
C5—C4—C6—C7	−178.89 (16)	C11—C12—C14—C15	−1.1 (3)

### Hydrogen-bond geometry (Å, º)

**Table:**

*D*—H···*A*	*D*—H	H···*A*	*D*···*A*	*D*—H···*A*
N4—H4*N*···O2^i^	0.91 (2)	1.91 (2)	2.7860 (17)	160.9 (17)
N2—H2*N*···O4^ii^	0.912 (19)	1.984 (19)	2.8872 (18)	170.4 (17)
C2—H2···O2^iii^	0.93	2.49	3.4082 (19)	171
C7—H7*A*···O3^ii^	0.96	2.54	3.458 (2)	159
C10—H10···O4^iv^	0.93	2.45	3.3563 (18)	164

Symmetry codes: (i) *x*−1/2, −*y*+1/2, *z*−1/2; (ii) *x*, *y*+1, *z*; (iii) −*x*, −*y*, −*z*+1; (iv) −*x*+1/2, *y*+1/2, −*z*+1/2.
